# Combined Transcriptome and Metabolome Analysis Reveals That the Potent Antifungal Pyrylium Salt Inhibits Mitochondrial Complex I in Candida albicans

**DOI:** 10.1128/spectrum.03209-22

**Published:** 2023-02-15

**Authors:** Quanzhen Lv, Lan Yan, Jinxin Wang, Jia Feng, Lu Gao, Lijuan Qiu, Wen Chao, Yu-Lin Qin, Yuanying Jiang

**Affiliations:** a School of Pharmacy, Naval Medical University, Shanghai, People’s Republic of China; b Shanghai Tenth People's Hospital, Tongji University School of Medicine, Shanghai, People’s Republic of China; c Fudan University Minhang Hospital, Shanghai, People’s Republic of China; Universidade de Sao Paulo

**Keywords:** *Candida albicans*, pyrylium salt, antifungal agents, oxidative phosphorylation, mitochondrial complex I

## Abstract

Based on the structural modification of SM21, xy12, a new pyrylium salt derivative with enhanced antifungal activities, was synthesized. The MICs (MIC_90_) of xy12 against Candida albicans ranged from 0.125 to 0.25 μg/mL, about 2-fold lower than those of SM21. In addition, xy12 inhibited hypha and biofilm formation in C. albicans in a dose-dependent manner. A total of 3,454 differentially expressed genes and 260 differential metabolites were identified in the xy12-treated C. albicans by RNA-seq and non-targeted metabolomics. By integrating KEGG pathway enrichment analysis, we found that inhibition of oxidative phosphorylation was the important antifungal mechanism of action of xy12. Electron transport through mitochondrial respiratory complexes I to IV is the common process of oxidative phosphorylation. Compared with the sensitivity of the wild-type SC5314 to xy12, decreased sensitivities in mitochondrial complex I (CI)-deficient mutants and increased sensitivities in mitochondrial complex III- and IV-deficient mutants suggested that the antifungal effects of xy12 were dependent on CI. Consistently, xy12 exhibited antagonism with rotenone, an inhibitor of CI, and significantly inhibited the expression and activity of CI. Meanwhile, the phenotypes in the xy12-treated C. albicans were similar to those in the CI-deficient mutants, such as decreased ATP production, reduced mitochondrial membrane potential, loss of mitochondrial DNA, inability to utilize nonfermentative carbon sources, and decreased cell wall N-linked mannoproteins. Collectively, our results revealed that the pyrylium salt xy12 could constrain oxidative phosphorylation by inhibiting mitochondrial complex I in C. albicans, providing a novel lead compound for the development of mitochondria-targeted antifungal drugs.

**IMPORTANCE** The development of new antifungal drugs is critical for solving the problem of antifungal resistance and expanding the limited variety of clinical antifungal drugs. Based on the modification of the pyrylium salt SM21, a new lead compound, xy12, was synthesized which was effective against *Candida* species both *in vitro* and *in vivo*. In this study, conjoined analysis of the transcriptome and metabolome elucidated the antifungal mechanism of action of xy12, which inhibited the activity of mitochondrial complex I in C. albicans. Targeting fungi-specific mitochondrial complex proteins has been reported as a promising antifungal strategy. Our study provided a new lead compound for targeting C. albicans mitochondrial complex I, which could be beneficial for discovering novel antifungal drugs.

## INTRODUCTION

*Candida*, Aspergillus, and Cryptococcus are the main opportunistic pathogens that cause invasive fungal infections in immunocompromised patients (1, 2). Under current antifungal treatments, invasive fungal infections cause about 1.6 million deaths each year, which is comparable to the number of deaths caused by tuberculosis (3). Additionally, fungal infections increase the lethality risk of other microbial infections. Statistics collected by Lansbury et al. (4) showed that the most common COVID-19-associated fungal infections are caused by C. albicans, which results in longer hospital stays and higher mortality (5). Because invasive candidiasis is a huge threat to human health, effective medical strategies are crucial to improve survival rates. Although current antifungal agents can cure most fungal infections, increasing resistance to azoles and echinocandins, the high toxicity of polyenes, and the poor oral bioavailability of polyenes and echinocandins have increased the need for novel antifungal compounds (6, 7).

As the powerhouse of eukaryotic cells, mitochondria are vital for energy metabolism, including anabolic and catabolic processes: for example, energy production, carbon, nitrogen, lipid, and iron metabolism, and the synthesis of amino acids, nucleic acids, and phospholipids (8). Mitochondria produce most of the cellular ATP needed for survival through the tricarboxylic acid (TCA) cycle and oxidative phosphorylation. The functional integrity of mitochondria is essential for the maintenance of C. albicans virulence (9). Defects in mitochondrial function, for instance, in ribosome synthesis, mitochondrial transcription, protein transport, or the function of the respiratory electron transport chain (ETC) could impair virulence. The significance of mitochondria is not limited to affecting metabolism. Many fungi-specific proteins have also been reported, which could be promising targets for the development of selective antifungals (10, 11). In recent years, the development of mitochondria-targeted inhibitors has made great progress. The arylamidine T-2307, which is currently in phase II clinical trials, can be efficiently internalized by C. albicans through polyamine transporters and inhibit complexes III and IV of the respiratory chain (12). The compound F901318, which targets mitochondrial dihydrolactate dehydrogenase, is also in phase II clinical research and is effective against Aspergillus infections (13). Ilicicolin-H, a polyketide, exhibits antifungal effects against Cryptococcus, *Candida*, and Aspergillus spp. through the inhibition of mitochondrial cytochrome bc_1_ reductase (14). All of these studies demonstrate that targeting mitochondria is an effective strategy for developing antifungal drugs (15).

SM21 is a cationic pyrylium compound with mitochondria-targeting properties. Wong et al. (16) discovered the strong antifungal activity of the lipophilic pyrylium salt SM21. However, its specific mechanism of action is still unclear. In our study, based on the structure of SM21, we obtained a new lipophilic pyrylium salt, xy12, which showed improved antifungal activity and reduced cytotoxicity both *in vitro* and *in vivo* (17). In the present study, integrated analysis of the transcriptome and metabolome revealed that oxidative phosphorylation could be suppressed by treatment with xy12. By using several mutants of the mitochondrial respiratory complexes and investigating mitochondrial functions, we further demonstrated that xy12 inhibits mitochondrial respiratory complex I in C. albicans. Therefore, our results provide a novel lead compound and strategy for antifungal drug development.

## RESULTS

### Compound xy12, derived from the modification of SM21, significantly inhibits growth and biofilm formation in *C. albicans*.

**FIG 1:**
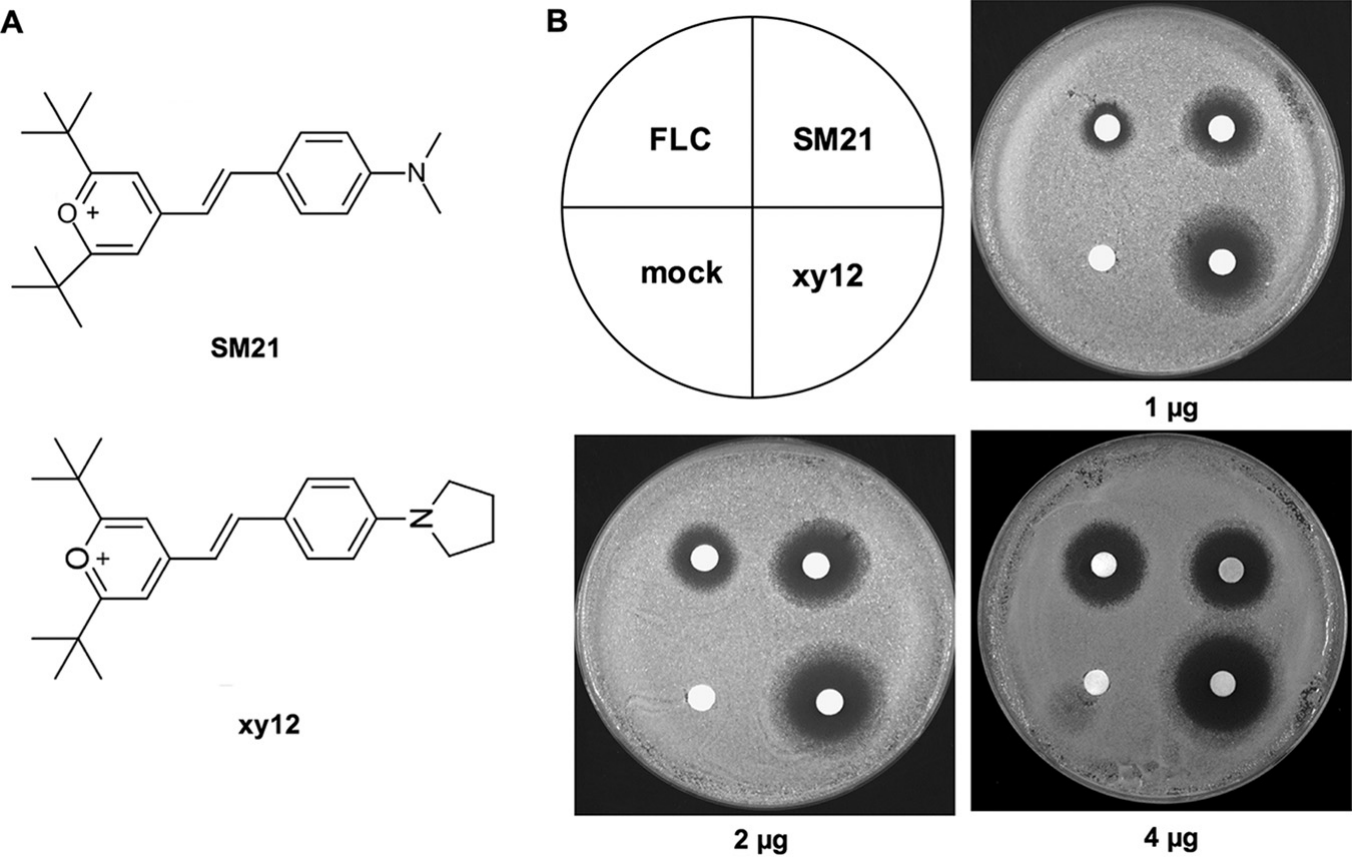
The chemical structures and antifungal activity of compounds SM21 and xy12. (A) The chemical structures of SM21 and xy12. (B) Filter disk-diffusion assay of Candida albicans SC5314. Filter disks with 1, 2, and 4 μg fluconazole, SM21, or xy12 were placed on yeast extract-peptone-dextrose (YPD) agar and cultured at 30°C for 24 h.

**FIG 2:**
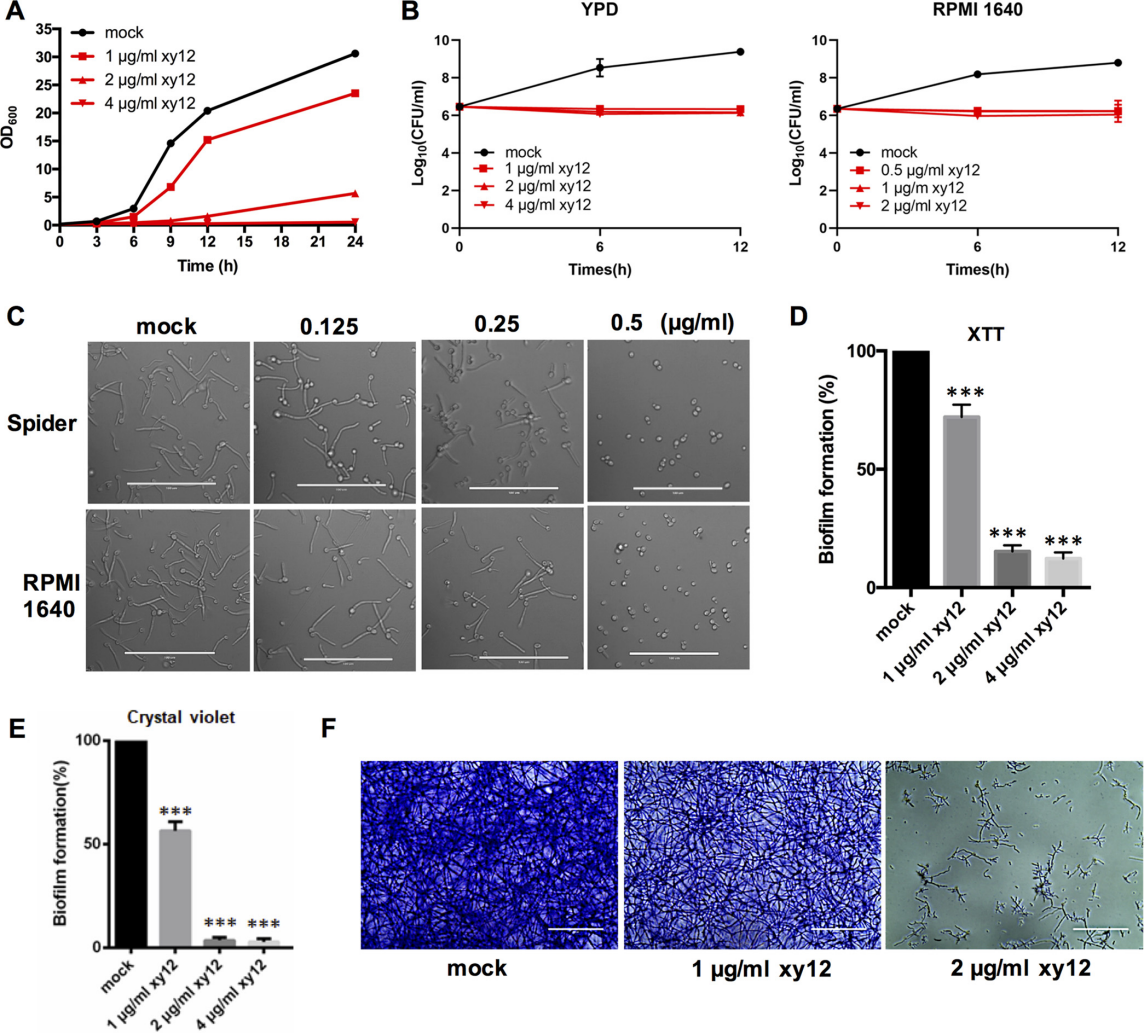
The compound xy12 inhibited growth and hypha and biofilm formation in C. albicans. (A) Time-growth curves. C. albicans SC5314 was treated with 1, 2, or 4 μg/mL of xy12 for 24 h and the OD_600_ (optical density at 600 nm) was measured at the indicated time points. (B) Time-kill curves of xy12 against C. albicans. C. albicans SC5314 was incubated with different concentrations of xy12 for 6 or 12 h in RPMI 1640 or YPD medium and plated on Sabouraud dextrose agar to count the living clones. (C) Hypha formation. C. albicans SC5314 was adjusted to 3 × 10^5^ cells/mL in Spider or RPMI 1640 medium with or without the indicated concentrations of xy12 and cultured at 37°C for 3 h. (D) Biofilm formation detected by XTT [2,3-bis-(2-methoxy-4-nitro-5-sulfophenyl)-2H-tetrazolium-5-carboxanilide] reduction. Biofilms of C. albicans SC5314 with or without xy12 treatment were induced in RPMI 1640 medium at 37°C for 24 h. XTT/menadione was added to the wells and the absorbance of supernatant was measured at 490 nm. ***, *P* < 0.001, unpaired *t* test. (E) Biofilm formation stained by crystal violet. The biofilms were stained with 0.4% crystal violet and quantified. (F) The coated cells were photographed.

Based on structural modification of the lipophilic pyrylium salt SM21, we obtained a new compound, xy12 (Fig. 1A). The MICs (MIC_90_) of xy12 against C. albicans ranged from 0.125 to 0.25 μg/mL, about 2-fold lower than the MIC_90_ values of SM21 (Table 1). In particular, the MIC_90_ of xy12 against the clinical isolates 103, 385, UCA3, and UCA42 ranged from 0.125 to 0.25 μg/mL, suggesting that xy12 was effective against fluconazole-resistant C. albicans. To further compare the antifungal activities of xy12 and SM21, 1, 2, and 4 μg of fluconazole, SM21, and xy12 was spotted in filter paper disks. The inhibition zones of xy12 were larger than those of SM21 and fluconazole at the same doses (Fig. 1B). Next, the time-growth curves in yeast extract-peptone-dextrose (YPD) medium were measured. We found that 1 μg/mL of xy12 slowed the growth of C. albicans SC5314, while 2 and 4 μg/mL of xy12 almost completely inhibited C. albicans growth within 24 h (Fig. 2A). In counting the number of live colonies on YPD and RPMI 1640 plates containing 0.5 to 4 μg/mL xy12, we found that xy12 showed no fungicidal activity within 12 h (Fig. 2B). Next, we investigated the antifungal activities of xy12 on hypha and biofilm formation. Hypha lengths became shorter with increasing concentrations of xy12. Almost all the C. albicans were reduced to yeast by treatment with 0.5 μg/mL of xy12 in both Spider and RPMI 1640 medium (Fig. 2C). Biofilms composed of hyphae and yeast cells are critical for drug resistance in C. albicans. Biofilm formation was measured by XTT [2,3-bis-(2-methoxy-4-nitro-5-sulfophenyl)-2H-tetrazolium-5-carboxanilide] reduction and crystal violet staining. Our results showed that about 30% to 40% of the biofilm was inhibited by 1 μg/mL xy12. Moreover, biofilm formation was almost completely inhibited to about 15% with 2 or 4 μg/mL of xy12 (Fig. 2D). Crystal violet staining showed that 1 μg/mL of xy12 significantly reduced the number of cells adhering to the bottom of the wells, leading to a looser biofilm and weaker crystal violet staining. C. albicans treated with 2 μg/mL of xy12 did not form a mature biofilm, and only a small number of yeast and hypha cells was observed at the bottom of each well (Fig. 2E and F). Collectively, these results indicated that xy12 has strong antifungal activities against C. albicans growth and biofilm formation.

**TABLE 1:** MICs of xy12, SM21, and FLC against C. albicans isolates^*a*^

C. albicans isolate	MIC_90_ (μg/mL)		
	xy12	SM21	FLC
SC5314	0.125	0.25	0.25
UCA32	0.125	0.25	0.5
UCA21	0.25	0.5	0.125
103	0.125	0.25	>64
385	0.25	0.25	>64
UCA3	0.125	0.25	>64
UCA42	0.25	0.5	>64

a FLC, fluconazole.

### Compound xy12 negatively regulated the expression of genes involved in mitochondrial oxidative phosphorylation.

**FIG 3:**
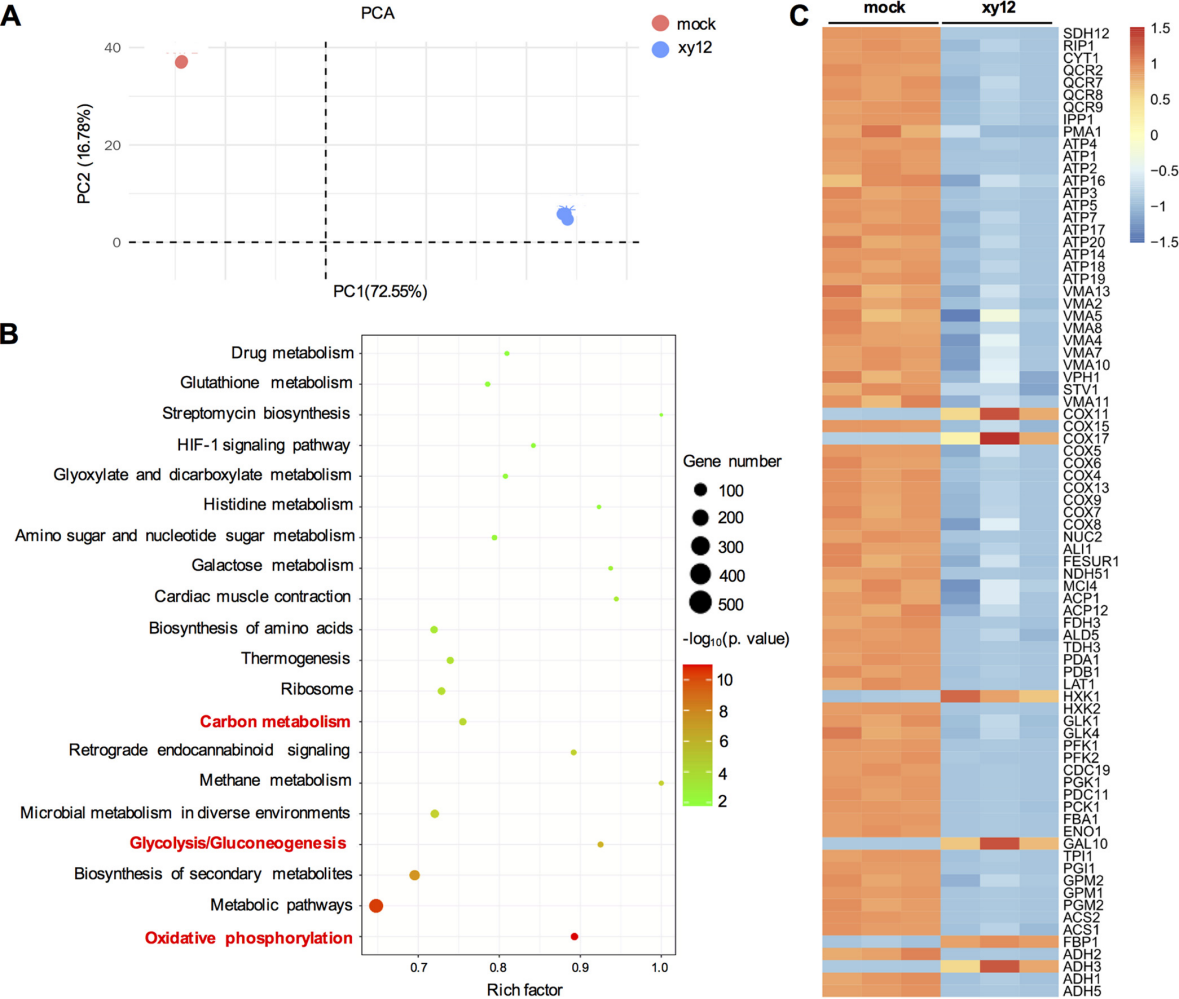
Effect of xy12 on the transcriptome in C. albicans. (A) Principal-component analysis of RNA-seq to evaluate the correlation and dispersion between different samples. The groups of dimethyl sulfoxide (DMSO)-treated (mock) and xy12-treated (xy12) C. albicans are shown in red and blue. (B) The top 20 enriched KEGG pathways are shown. Vertical axis represents KEGG annotation, horizontal axis represents the Rich factor. (C) Heatmap of differentially expressed genes (|log_2_-fold change| ≥ 1, *P* < 0.05) involved in oxidative phosphorylation. Blue color indicates downregulated genes.

To examine the antifungal mechanisms of action of xy12, genome-wide expression profiles in cells treated with xy12 were detected by RNA-seq. Principal-component analysis (PCA) was used to assess differences between the two groups and biological replication of samples within a group. As shown in Fig. 3A, the samples in the mock- and xy12-treated groups were scattered and samples in each group were aggregated, which indicated the high quality of RNA-seq. Overall, a total of 1,826 genes were upregulated and 1,628 genes were downregulated. A Gene Ontology (GO) term analysis determined that the top 10 biological processes influenced by xy12 were all related to metabolism, mostly including organonitrogen compound biosynthetic processes, small molecule metabolism, nucleotide metabolism, etc. In the analysis of molecular function and cellular composition, proton transmembrane transporter activity, NADH dehydrogenase activity, proton-transporting ATP synthase complex, and respiratory chain complexes were significantly enriched (Fig. S1). Moreover, KEGG analysis also enriched differentially expressed genes (DEGs) in different metabolic pathways, such as carbon metabolism, glucose metabolism, amino acid metabolism, and oxidative phosphorylation (Fig. 3B). Among these, oxidative phosphorylation is the key process in the metabolism of carbohydrates, amino acids, and lipids to generate ATP under aerobic conditions (18). Thus, many transcriptional changes may be due to altered ATP production disrupted by xy12, resulting in the activation or inhibition of various signaling pathways. Through the enriched pathways, we speculated that the effects of xy12 on nutrient metabolism were dependent on its inhibition of the common oxidative phosphorylation process. Oxidative phosphorylation is performed by the electron transport chain (composed of mitochondrial respiratory complexes I to V) and chemiosmosis. The genes involved in oxidative phosphorylation were then selected and shown as a heatmap (Fig. 3C). Among these, only 6 genes, including *COX11*, *COX17*, *HXK1*, *GAL10*, *FBP1*, and *ADH3*, were upregulated in the xy12-treated group. A total of 73 genes were downregulated, most of which encode mitochondrial complexes I (*MCI4*, *NDH51*), II (*SDH2*, *SDH12*, *RIP1*, *CYT1*), III (*QCRs*), IV (*COXs*) and V (*ATPs*, *VMA2*, *PMA1*). Overall, transcriptomic analysis of C. albicans suggested that compound xy12 could change the metabolism of various nutrients by suppressing the expression of oxidative phosphorylation genes.

### Carbohydrate metabolites were changed in response to xy12 treatment.

**FIG 4:**
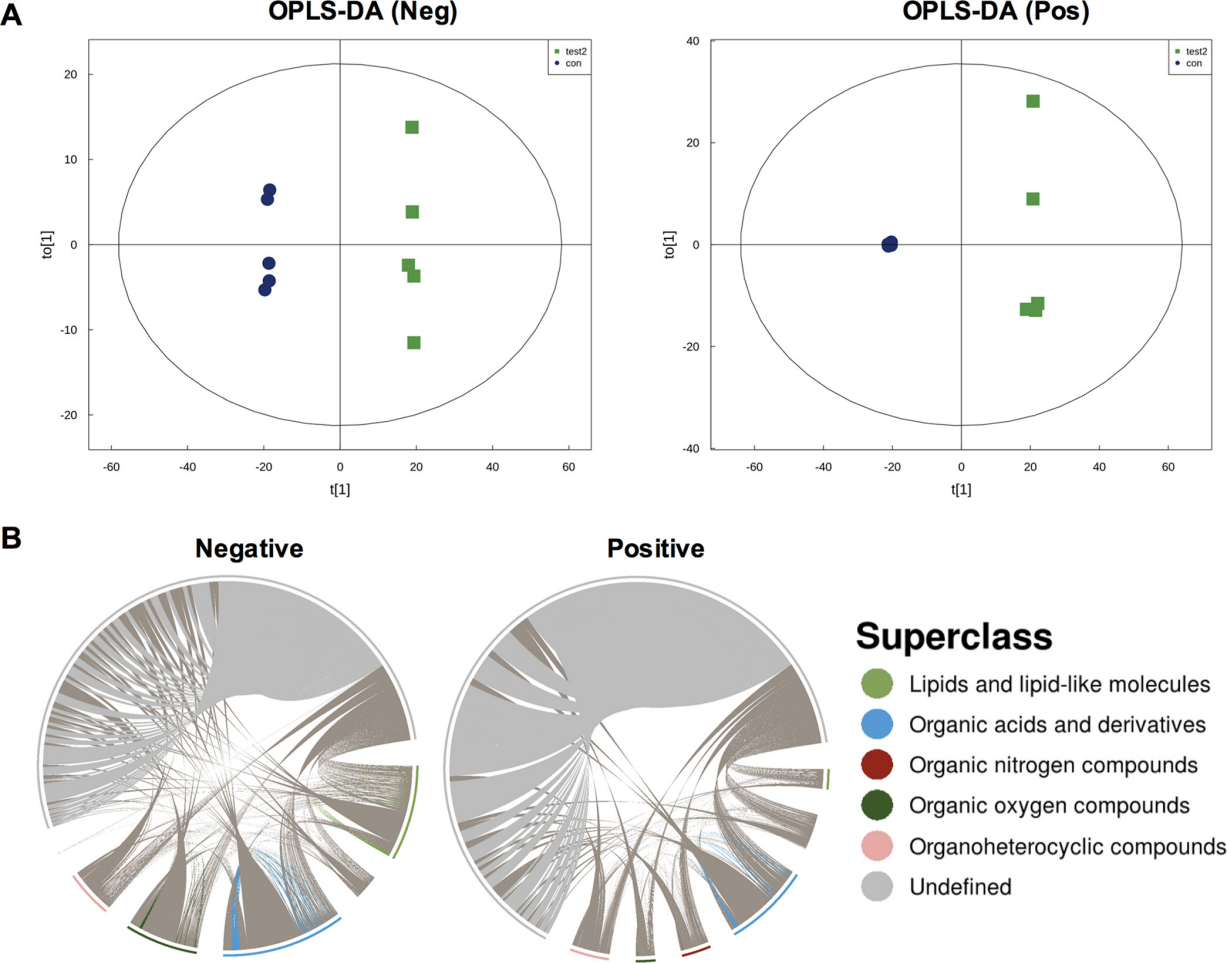
Metabolomics analysis of samples extracted from xy12-treated C. albicans. (A) The relationship between metabolite content and groups was analyzed by orthogonal partial least-squares discriminant analysis. Scores plot showed the groups of xy12-treated (blue) and DMSO-treated (green) C. albicans SC5314 in a negative (left) and positive (right) ion model. (B) Correlations between metabolites shown by chord diagrams. Arcs on the outer circle indicate the categories of significantly different metabolites. Colored lines indicate the correlations within the various metabolites.

**FIG 5:**
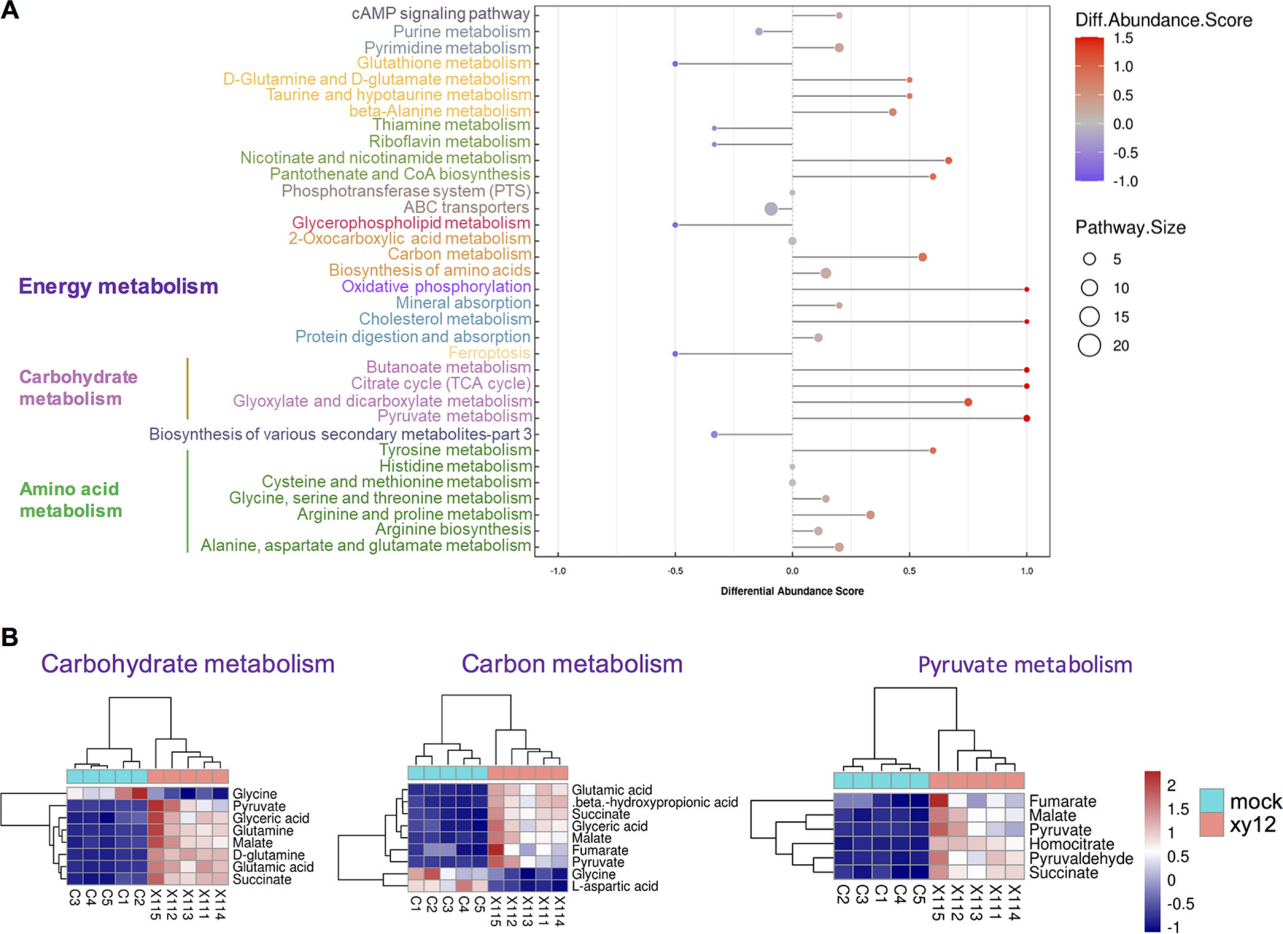
Treatment with xy12 changed the metabolites in carbohydrate and energy metabolism pathways. (A) Differential abundance (DA) score analysis of metabolites. The DA score represents the total change of all metabolites in the metabolic pathway. A score of 1 indicates that all identified metabolites in the pathway were upregulated; a score of of −1 indicates that all metabolites were downregulated. Dot size represents the number of metabolites in a pathway. (B) Heat map of differential metabolites located in the carbohydrate metabolism, carbon metabolism, and pyruvate metabolism pathways. Z-score standardized peak intensity was used to portray the relative quantity and changing trend of each metabolite in heat maps by MeV software. Red indicates upregulation, blue indicates downregulation.

Based on the changed expression of genes encoding nutrient metabolism and oxidative phosphorylation, non-targeted metabolomics were used to further investigate the effects of xy12 on metabolism in C. albicans. By combining the positive and negative ions, a total of 1,197 metabolites were identified in C. albicans treated with xy12. In testing with an orthogonal partial least-squares discriminant analysis (OPLS-DA) multiple pattern recognition model, metabolic profiling showed obvious differences between xy12-treated and untreated cells (Fig. 4A). In the OPLS-DA analysis, significant metabolites were defined as having variable importance in projection (VIP) > 1 and *P* < 0.05. A total of 260 differential metabolites were detected in the xy12-treated C. albicans (Table S2 and S3). The correlation analysis of significantly different metabolites was shown as chord diagram, which is beneficial for understanding the metabolic classification and reciprocal regulatory relationship between metabolites. As shown in Fig. 4B, organic acids and derivatives accounted for the highest proportion, and the correlation between organic acids was significant. Because organic acids and derivatives are mainly involved in the processes of carbohydrate and amino acid metabolism, our results were consistent with the transcriptomic results. Next, to investigate the correlation between metabolites and intracellular signaling pathways, KEGG pathway annotation and analysis were performed on the significantly different metabolites. As shown in Fig. 5A and Fig. S2, the differential abundance score indicated that most metabolic pathways were upregulated and only 2 pathways were downregulated. A large number of metabolites, such as alanine, aspartate, arginine, and histidine, were enriched in amino acid metabolism, but the upregulated abundance score was not high. By comparing differential abundance scores, we found that carbohydrate metabolites were the most significantly upregulated pathways, including pyruvate metabolism, glyoxylate and dicarboxylate metabolism, the citrate cycle, and butanoate metabolism. Differential metabolites in the carbohydrate, carbon, and pyruvate metabolism pathways were displayed as heatmaps. As shown in Fig. 5B, the intermediate products of glucose metabolism, such as glutamine, pyruvate, malate, fumarate, and succinate, were significantly increased in C. albicans treated with xy12. Among these, glutamine can be converted into α-ketoglutarate, which is then metabolized into pyruvate and enters the tricarboxylic acid cycle. Other metabolites, such as pyruvate, malate, fumarate, and succinate, are intermediates in the tricarboxylic acid cycle, whose normal operation often requires the conversion of NADH to NAD^+^ through the electron transport chain. Accumulation of pyruvate, malate, fumarate, and succinate in the xy12-treated C. albicans indicated that the antifungal effects of xy12 may depend on the perturbation of the tricarboxylic acid cycle or electron transport chain.

### Integration of transcriptome and metabolome analysis confirmed the inhibition of xy12 on *C. albicans* oxidative phosphorylation.

**FIG 6:**
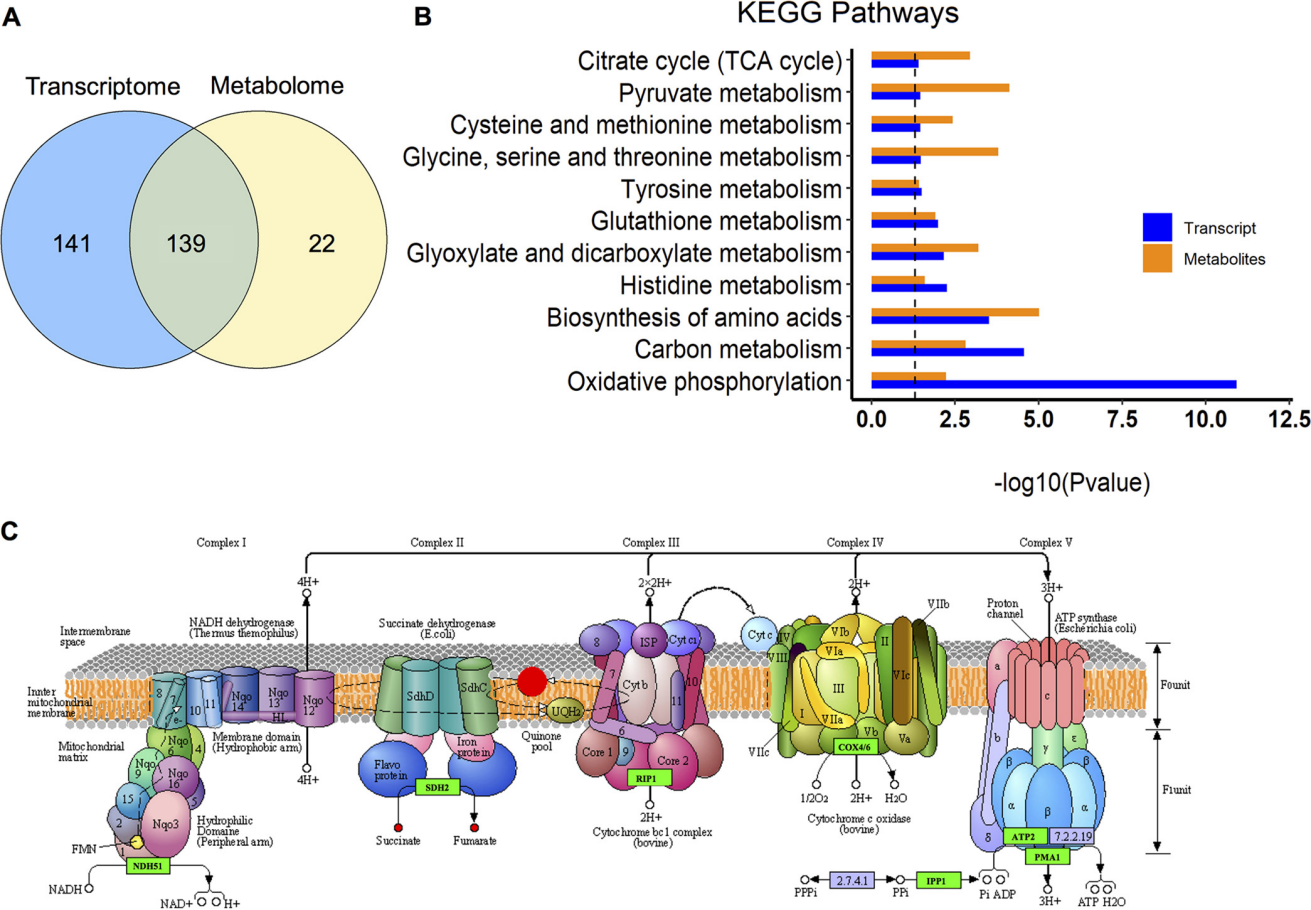
Combined analysis of the metabolome and transcriptome of C. albicans treated with xy12. Quantity and description of common enriched KEGG pathways in transcriptome and metabolome (A). Blue represents the transcriptome and orange represents the metabolome. Horizontal axis indicates the significance of enrichment (B). (C) Schematic representation of oxidative phosphorylation pathway. Green boxes represent the downregulated genes, red circles represent the upregulated metabolites.

Currently, the most common analysis of integrated transcriptome and metabolome is based on the same enriched KEGG pathways. As shown in Fig. 6A, there were 139 common KEGG pathways significantly enriched in the transcriptome and metabolome in the xy12-treated group. Only 22 pathways were enriched in the metabolome, but not in the transcriptome, indicating good consistency between the transcriptomics and metabolomics analyses. The top 13 pathways, according to the enrichment significance of genes and metabolites, are shown in Fig. 6B. Obviously, oxidative phosphorylation and the carbon source metabolism pathway are ranked in the top two. Under aerobic conditions, fermentative carbon sources generate ATP through three processes: glycolysis, the tricarboxylic acid cycle, and oxidative phosphorylation. In the schematic diagram of oxidative phosphorylation, the expression of genes encoding mitochondrial complexes (marked by the green box) was significantly downregulated, and metabolites such as succinate and fumarate (red dots) were significantly accumulated, showing the inhibition of oxidative phosphorylation more intuitively (Fig. 6C). Therefore, the common enriched pathways suggested that xy12 mainly inhibited oxidative phosphorylation, which in turn led to the accumulation of upstream metabolites such as fumarate and succinate in the tricarboxylic acid cycle.

### The antifungal activity of xy12 was partially dependent on the inhibition of mitochondrial complex I.

**FIG 7:**
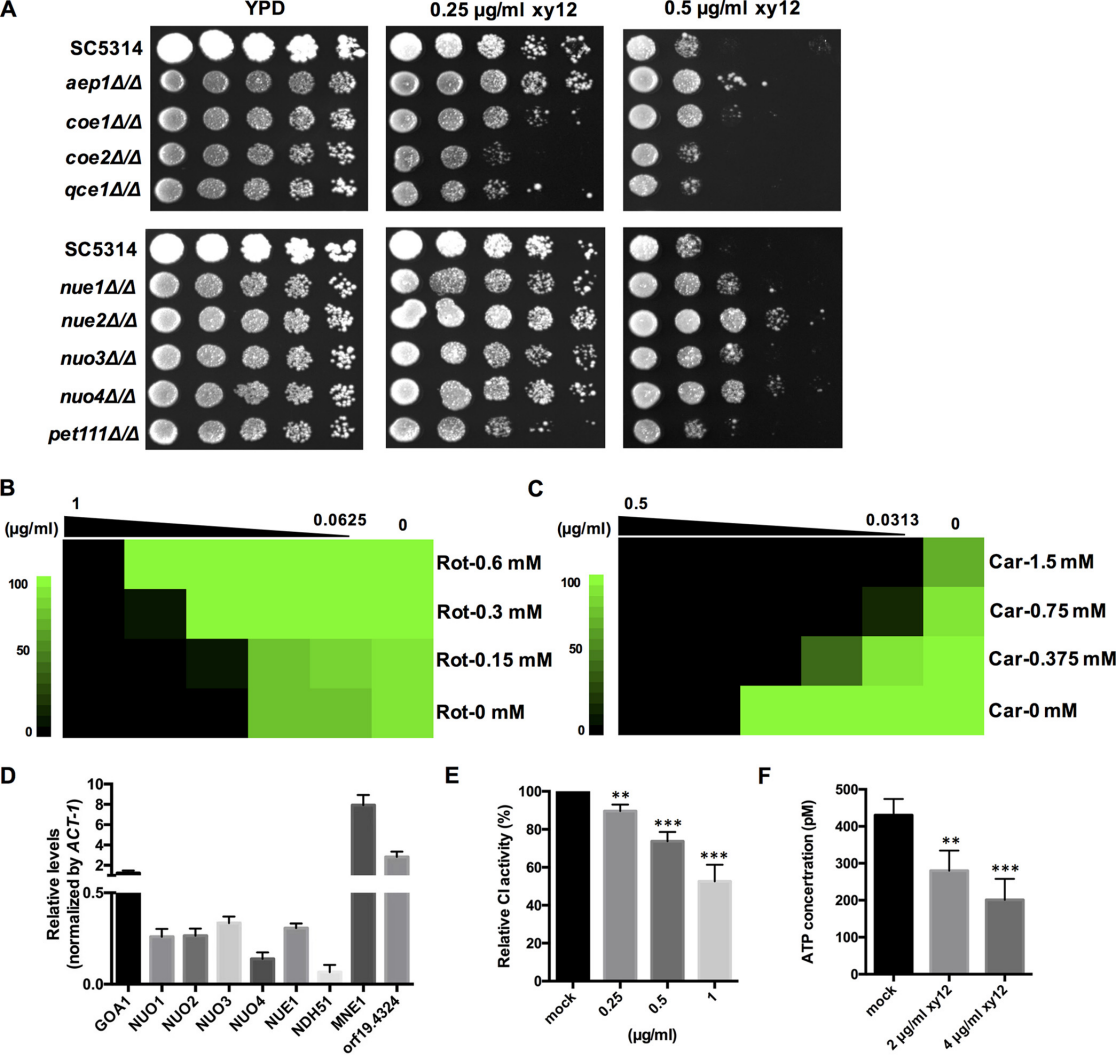
Effects of xy12 on the mitochondrial complexes and ATP production. (A) Sensitivities of the mitochondrial complex-deficient mutants to xy12 as determined by spot assay. C. albicans mutants were spotted on YPD agar with the indicated concentrations of xy12 and cultured for 48 h. (B and C) Checkerboard assays of the antifungal activity of rotenone (B) and carboxin (C) combined with xy12 against C. albicans SC5314 for 48 h. Growth inhibition was determined by OD_600_. Green regions represent higher cell density. (D) Relative expression of genes encoding mitochondrial complex I determined by reverse transcription-quantitative PCR (RT-qPCR). Total RNA was extracted from C. albicans SC5314 treated with 2 μg/mL of xy12 for 3 h. The expression of each gene was normalized to *ACT1* and calculated by the threshold cycle (2^−ΔΔ^*^CT^*) method. Relative levels of mRNA represented the fold changes between the xy12-treated cells and the DMSO-treated cells (mock). The gene expression of DMSO-treated cells was normalized to 1. (E) The activity of mitochondrial complex I inhibited by xy12. Mitochondria were isolated from C. albicans SC5314 and treated with 0.25, 0.5, and 1 μg/mL of xy12 for 3 h. (F) Intracellular ATP content of C. albicans SC5314 treated with xy12 for 6 h. ATP content was measured by the Titer-Glo cell reagent. Data are shown as mean ± standard deviation. **, *P* < 0.01; ***, *P* < 0.001 by unpaired *t* test.

The process of oxidative phosphorylation is accompanied by electron transport via the ETC, located in the inner mitochondrial membrane (19). Considering the enrichment of oxidative phosphorylation in the transcriptome and metabolome, as mentioned above, the sensitivity of mitochondrial complex-deficient C. albicans mutants to xy12 was determined by spot assay. The mitochondrial complex-deficient mutants used in our studies included *NUE1*-, *NUE2*-, *NUO3*-, and *NUO4*-disrupted mutants (encoding mitochondrial complex I subunits), a *QCE1*-disrupted mutant (encoding mitochondrial complex III subunits), and *COE1*-, *COE2*-, *AEP1*-, and *PET111*-disrupted mutants (encoding mitochondrial complex IV). As shown in Fig. 7A, compared with C. albicans SC5314, the mitochondrial complex IV-deficient *coe1*Δ/Δ *and coe2*Δ/Δ mutants were slightly and obviously sensitive, respectively, to 0.25 μg/mL of xy12. However, the disruption of other genes encoding mitochondrial complex IV, such as *AEP1* and *PET111*, had no significant influence on the sensitivity of C. albicans. For mitochondrial complex III, the disruption of *QCE1* slightly increased C. albicans sensitivity to 0.25 μg/mL of xy12. However, in C. albicans with the disruption of genes encoding mitochondrial complex I, *nue1*Δ/Δ, *nue2*Δ/Δ, *nuo3*Δ/Δ, and *nuo4*Δ/Δ mutants showed various degrees of resistance to 0.5 μg/mL of xy12, as the colonies of CI mutants grew more than those of C. albicans SC5314. Mitochondrial complex I is located upstream from complexes III and IV (Fig. 6C). Based on the decreased sensitivity of the complex I-deficient mutants and increased sensitivities of the complex III- and IV-deficient mutants, we speculated that xy12 mainly inhibits the function of mitochondrial complex I. Therefore, the antifungal effect of xy12 was attenuated in the mitochondrial complex I-deficient mutants. To further verify the inhibitory effect of xy12, we determined the influences of rotenone, an inhibitor of mitochondrial complex I, and carboxin, an inhibitor of complex II, on xy12 activity. As expected, 0.3 or 0.6 mM rotenone alone could barely inhibit the growth of C. albicans but increased the MIC_90_ of xy12 from 0.25 to 0.5 and 1 μg/mL, respectively, showing an antagonistic effect with xy12 (Fig. 7B). In contrast, 0.375 to 1.5 mM carboxin reduced the MIC_90_ of xy12 from 0.25 to 0.125 or even 0.0313 μg/mL, being synergistic with xy12 (Fig. 7C). These results indicated that compound xy12 may exert antifungal activity by inhibiting mitochondrial complex I.

To examine the changes of mitochondrial complex I, the expression level of the encoding genes was detected by reverse transcription-quantitative PCR (RT-qPCR). As shown in Fig. 7D, the expression of *NUOs*, *NUE1*, and *NDH51*, which encode the NADH-ubiquinone oxidoreductase subunits, were significantly downregulated. Meanwhile, the mRNA levels of *MNE1* and *ORF19.4324*, which are required for the expression, assembly, and membrane localization of complex I, were upregulated as feedback. Furthermore, the C. albicans mitochondria was isolated and the effect of xy12 on the activity of mitochondrial complex I was examined *in vitro*. The addition of 0.5 or 1 μg/mL of xy12 reduced the activity of mitochondrial complex I by about 30% and 50%, respectively, suggesting a direct inhibitory effect of xy12 on mitochondrial complex I (Fig. 7E). Because the process of oxidative phosphorylation is critical for mitochondrial ATP production, the intracellular ATP content was further investigated. With the treatment of 2 or 4 μg/mL of xy12, ATP production levels were significantly reduced compared to those in the mock-treatment group (Fig. 7F). In conclusion, compound xy12 reduced intracellular ATP production by inhibiting oxidative phosphorylation, especially the activity of mitochondrial complex I, in C. albicans.

### Compound xy12 disrupted mitochondrial morphology and function in *C. albicans*.

**FIG 8:**
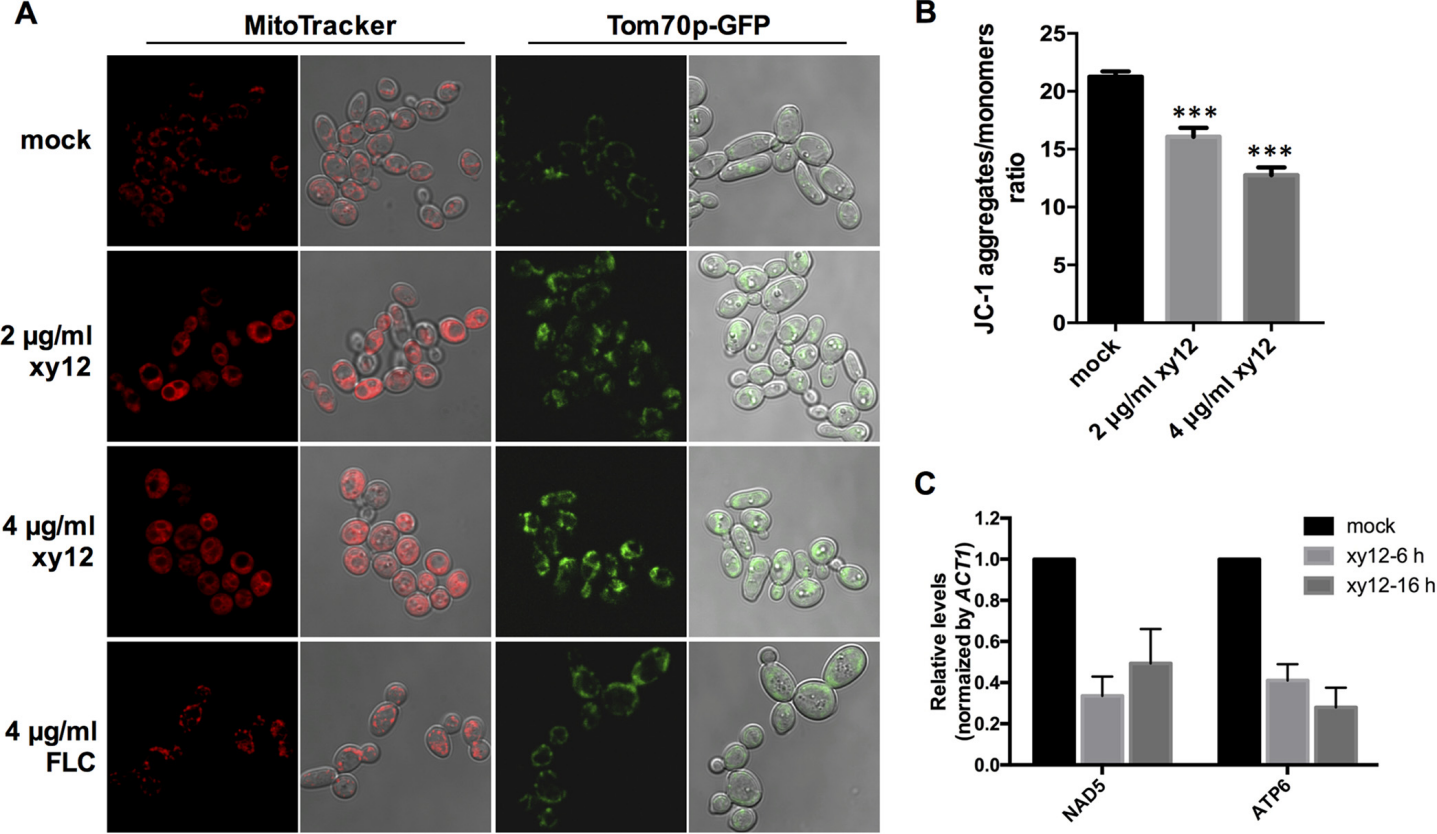
Abnormal mitochondrial morphology and functions caused by xy12. (A) Mitochondrial morphology of C. albicans SC5314 or Tom70p-GFP strains treated with xy12 or fluconazole (FLC) was observed. C. albicans SC5314 was stained with 100 nM MitoTracker Red CMXRos for 30 min. (B) Mitochondrial membrane potential of C. albicans SC5314 was measured by JC-1. C. albicans were stained with JC-1 buffer for 20 min and the fluorescence of JC-1 was detected at 590 nm and 530 nm. (C) Mitochondrial DNA (mtDNA) of C. albicans SC5314 was analyzed by RT-qPCR. Total DNA of C. albicans was extracted and the relative expression of mtDNA *NAD5* and *ATP6* was normalized to that of *ACT1*.

As dynamic organelles, mitochondria constantly undergo fission and fusion. Mitochondrial abnormal remodeling has been observed in C. albicans cells treated with the respiration inhibitor sodium nitroprusside, the alternative oxidase inhibitor salicylhydroxamic acid, the complex I inhibitor rotenone, or the complex II inhibitor thenoyltrifluoroacetone (20, 21). To investigate whether the inhibition of oxidative phosphorylation by xy12 influences mitochondrial morphology, we used the mitochondria-specific dyes MitoTracker Red and Tom70p-GFP (localized at the mitochondrial membrane). As shown in Fig. 8A, mitochondria in normal and fluconazole-treated C. albicans strains were linearly distributed, whereas mitochondria in xy12-treated cells were diffusely distributed within the cellular plasma, suggesting that the compound xy12 impaired mitochondrial fission and fusion. In addition to morphogenesis, reduction of mitochondrial membrane potential is a common and dangerous situation associated with impaired oxidative phosphorylation (22). By measuring the JC-1 ratio of aggregate to monomer fluorescence, we found that the mitochondrial membrane potential of xy12-treated C. albicans strains decreased significantly (Fig. 8B). Moreover, by determining the levels of mitochondrial genes *NAD5* and *ATP6* through RT-qPCR, we found that mitochondrial DNA (mtDNA), which was maintained by some oxidative phosphorylation subunits, were also significantly reduced by the action of xy12 (Fig. 8C) (23). Overall, the impaired mitochondrial morphology, reduced mitochondrial membrane potential, and loss of mtDNA all confirmed that compound xy12 could inhibit mitochondrial complex I in C. albicans.

### Carbon source utilization and cell wall N-linked mannoprotein biosynthesis in *C. albicans* was inhibited by compound xy12.

**FIG 9:**
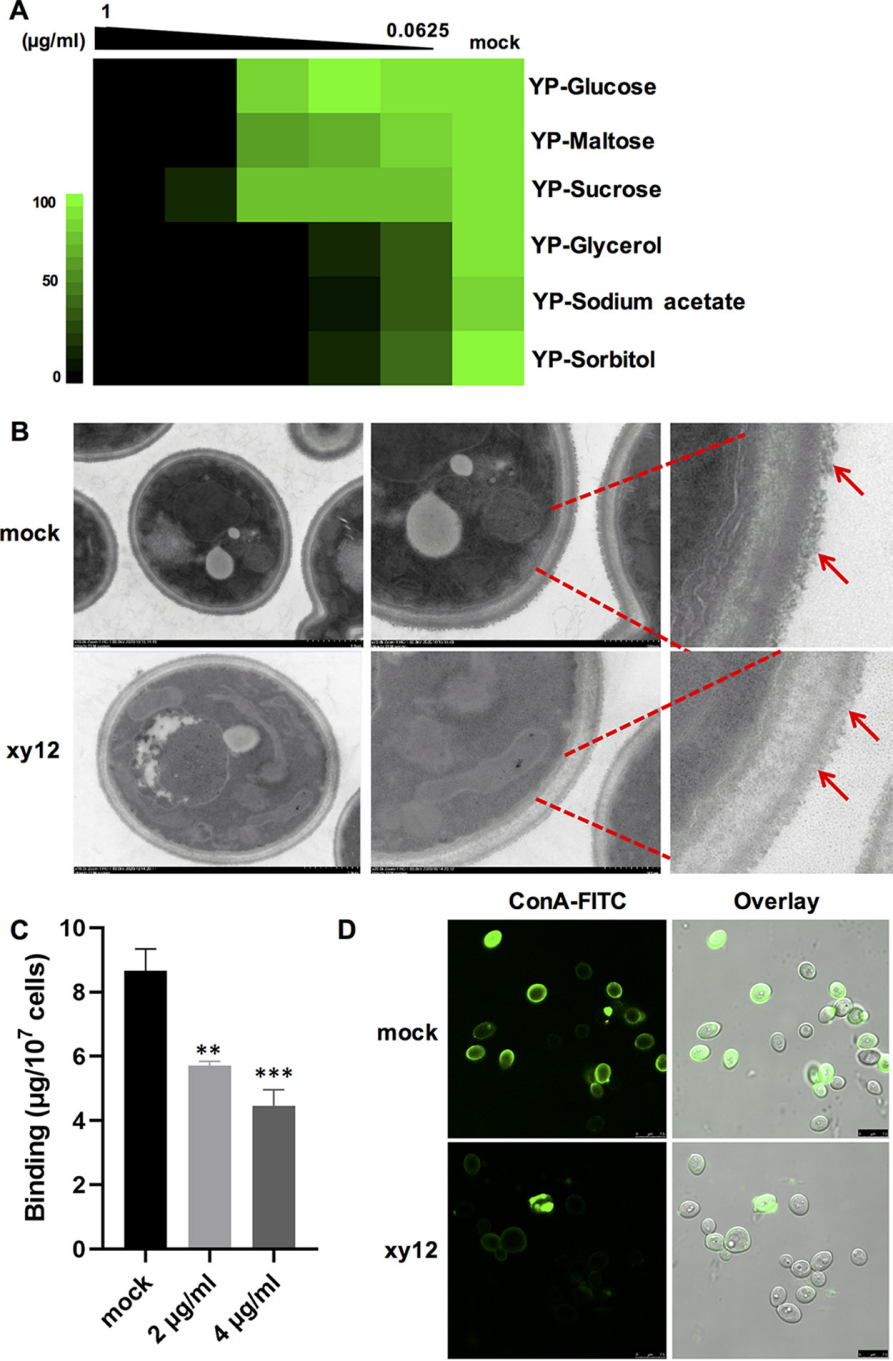
Abnormal carbon utilization and cell wall structures caused by xy12 treatment. (A) Checkerboard assays of antifungal activity of xy12 against C. albicans SC5314 in media containing different carbon sources. After 24 h of incubation, the OD_600_ was used to calculate growth inhibition. Green regions indicate higher cell density. (B) The cell wall of C. albicans SC5314 was observed by transmission electron microscopy. C. albicans was treated with 2 μg/mL of xy12 for 16 h in YPD medium. Red arrows indicate the cell wall fibers. (C) Phosphomannan of *C. albcians* SC5314 was stained by Alcian blue. C. albicans was treated with 2 or 4 μg/mL of xy12 for 16 h in YPD medium. Data are shown as mean ± standard deviation. **, *P* < 0.01; ***, *P* < 0.001 by unpaired *t* test. (D) Mannan of the cell wall was stained by ConA-FITC (fluorescein isothiocyanate). C. albicans SC5314 was treated with 4 μg/mL of xy12 for 16 h in YPD medium and photographed by confocal microscopy.

Sun et al. (10) found that mitochondrial complex I deletion mutants (*goa1*Δ/Δ, *nue1*Δ/Δ, *nue2*Δ/Δ, *nuo3*Δ/Δ, and *nuo4*Δ/Δ) were unable to grow on nonfermentable carbon sources or transition from yeast to hyphae. The inhibition of hypha and biofilm formation by xy12 is illustrated in Fig. 2. Here, we mainly focused on xy12’s inhibitory effects on carbon source utilization and cell wall structures. Non-fermentable carbon sources have been reported to force C. albicans to utilize the mitochondrial complexes for ATP production (24). Therefore, mitochondrial complex I-deficient C. albicans could not grow in medium only containing nonfermentable carbons. As expected, in medium containing yeast extract and peptone with the addition of 2% fermentation carbon sources, such as glucose, maltose, and sucrose, the MIC_90_ of xy12 against C. albicans were about 0.5 μg/mL. In contrast, with the addition of nonfermentable carbon sources, such as glycerol, sodium acetate, and sorbitol, the MIC_90_ of xy12 was lower than 0.125 μg/mL (Fig. 9A). These data suggested that compound xy12 reduced C. albicans growth more effectively in nonfermenting carbon sources. She et al. (25) also confirmed that mitochondrial complex I was closely integrated with cell wall polysaccharide synthesis and cell wall formation. They found that *GOA1*, which encodes a mitochondrial complex I accessory protein, was essential for the biosynthesis of cell wall N-linked mannoprotein, so we further examined the inhibitory effect of xy12 on the cell wall. As shown in Fig. 9B, treatment with xy12 led to sparser fibrils compared to those in normal cells, as observed by transmission electron microscopy, which is consistent with the requirement of mitochondrial complex I for cell wall integrity (25). Alcian blue is a cationic dye which binds to the negatively charged phosphomannan distributed on the surface of C. albicans. In our results, Alcian blue staining was significantly reduced in C. albicans treated with 2 or 4 μg/mL of xy12 (Fig. 9C). Subsequently, concanavalin A was used to specifically detect the C. albicans mannan. Using confocal imaging, we clearly observed that the fluorescence of xy12-treated C. albicans was much weaker than that of the control cells stained with ConA-FITC (fluorescein isothiocyanate) (Fig. 9D). These results further confirmed that compound xy12 could inhibit the biosynthesis of cell wall N-linked mannoproteins. Overall, the similar phenotypes in the xy12-treated C. albicans and the mitochondrial complex I-deficient mutants suggested that xy12 exerts antifungal effects through inhibiting mitochondrial complex I.

## DISCUSSION

The mortality rate of systemic candidiasis remains as high as 40% under current antifungal treatments (26). The synthesis of new structural antifungal compounds and elucidation of their antifungal mechanisms are urgently needed. In this study, through structural modification of SM21, we synthesized a compound, xy12, with stronger activity and lower toxicity (17). The skeleton structure of SM21 is pyrylium. As reported previously, fluorescent styrylpyrylium salts have been used for mitochondrial localization and visualization, indicating the mitochondria-targeting characteristics of pyrylium (27). In addition, a novel di-benzene-pyrylium-indolene was defined as an inhibitor of lipid II inhibitor, an essential component of bacterial cell wall synthesis, and showed effective activity against *Enterococcus* spp. *in vitro* and *in vivo* (28). Furthermore, the inhibition effects of benzopyrylium salts on breast tumor cells and Staphylococcus aureus have already been confirmed, but their mechanism of action is still not clear (29). The antifungal effect of pyrylium salt, SM21, was first screened by Wong et al. (16) Subsequent studies demonstrated that SM21 treatment results in compromised mitochondria and that SM21-resistant strains showed enhanced transcriptomic responses for peptide and protein metabolism. The MIC_90_ of SM21 against C. albicans increased 2-fold in medium supplied with peptone (30). In the present study, transcriptome and metabolome analysis indicated that xy12 significantly affected oxidative phosphorylation. Through subsequent phenotypic validation, we revealed that the critical mechanism of action of the compound xy12 is inhibiting mitochondrial complex I in C. albicans, leading to insufficient ATP production and disturbance of carbohydrate and amino acid metabolism. Meanwhile, xy12 also disrupted mitochondria morphology. Using MitoTracker staining, we found that the mitochondria of C. albicans showed a diffuse and nonlinear distribution with xy12 treatment. Because the disruption of mitochondrial membrane potential would influence the distribution of MitoTracker, we used Tom70p-GFP to further confirm this mitochondrial morphology, which also showed disrupted phenotypes. However, whether xy12 affects mitochondrial fusion or division has not been determined, and we will focus on this issue in our follow-up studies.

In the transcriptome, a total of 1,826 upregulated genes and 1,628 downregulated genes were detected. Many of these differentially expressed genes may be related to the metabolic disorders caused by xy12-disrupted ATP production. Autophagy is important in the tolerance of pathogenic fungi to nutrient starvation (31). In a GO term analysis, many upregulated genes were associated with pathogenesis and autophagy, suggesting that the xy12 treatment caused intracellular nutrient and energy starvation. Consistently, downregulated genes were associated with organonitrogen compound biosynthesis, small molecule metabolism, and ribonucleotide metabolism, suggesting the metabolic influence of xy12. In the subsequent signaling pathway enrichment analysis, the oxidative phosphorylation pathway was significantly enriched. Oxidative phosphorylation is critical for energy metabolism in aerobic fungi (32), which is required for the oxidation of carbohydrates, amino acids, and fats to produce ATP. Comprehensive downregulation of genes involved in oxidative phosphorylation reduced ATP production and may alter the expression of other genes to adapt to intracellular ATP starvation.

To clarify the changes in nutrient metabolism, we used untargeted metabolomics to further identify a total of 260 differential metabolites by negative and positive ion modes. Most of these differential metabolites were involved in amino acid, carbohydrate, and lipid metabolism. Among these, the amino acid and carbohydrate metabolism pathways were dominant, while lipid metabolism pathways were less dominant. This may be due to the use of high-pressure liquid chromatography-mass spectrometry (HPLC-MS) instead of gas chromatography-mass spectrometry (GC-MS) in our study, and thus the identified substances were dominated by highly polar metabolites. Because only a few lipids were detected, the related pathways were consequently not enriched. In general, correlated analyses of the transcriptome and metabolome revealed oxidative phosphorylation as the most obvious enriched pathway, which further shows the importance of oxidative phosphorylation in the antifungal mechanism of action of xy12.

Oxidative phosphorylation takes place in the mitochondria and produces ATP. It involves the oxidation-reduction process and generation of an electrochemical gradient by the electron transport chain. The electron transport chain is shared by the processes of carbohydrate, amino acid, and lipid oxidation, consisting of mitochondrial complexes I to V. Under aerobic conditions, electrons also pass through the mitochondrial complexes to generate ATP (33). The function of the mitochondrial complex has been verified by many knockout mutants. Mitochondrial complex mutants display deficiencies in growth, carbon source utilization, cell membrane and cell wall composition, yeast-to-hyphae transformation, biofilm formation, antifungal sensitivity, and interactions with host cells (24, 34). Some unique genes encoding mitochondrial complex subunits were identified as fungi-specific and were identified as potential antifungal targets. By examining the sensitivity of C. albicans with disruptions in genes encoding mitochondrial complexes, we preliminarily verified the antifungal mechanism of action of xy12. The sensitivities of mitochondrial complex I-deficient mutants (*nue1*Δ/Δ, *nue2*Δ/Δ, *nuo3*Δ/Δ, and *nuo4*Δ/Δ) to 0.5 μg/mL of xy12 were significantly decreased in comparison to the wild-type stain, suggesting that xy12 exerts antifungal effects by inhibiting mitochondrial complex I. Meanwhile, the disruption of mitochondrial complexes III or IV resulted in increased sensitivity to xy12, which may be due to the synergistic effect with the deficient mitochondrial complex I inhibited by xy12. However, the structure of C. albicans mitochondrial complex I (NADH:ubiquinone oxidoreductase) is complex, composed of 39 subunit proteins (35). Mutants with disruptions in different genes encoding mitochondrial complex I subunits showed different phenotypes. For example, transcriptional profiling of the *goa1*Δ/Δ, *nuo1*Δ/Δ, *nuo2*Δ/Δ, and *ndh51*Δ/Δ mutants displayed overlapping changes and subunit-specific functional assignments (11). In our study, the deletion of genes encoding different subunits of the same mitochondrial complex produced different sensitivities to xy12, which may be related to the specific functions of each subunit. Subsequently, we tested the activity of mitochondrial complex I *in vitro* and confirmed that the mitochondrial morphological and functional damage caused by xy12 was similar to that in mitochondrial complex I-deficient mutants. Mitochondrial complex-deficient mutants have a common phenotype that cannot utilize nonfermentative carbon sources. Unsurprisingly, we found that the MICs of xy12 against C. albicans were reduced 4 to 8 folds in media only containing nonfermenting carbon sources, which was more obvious than the effect of peptone found by Wong et al. (16). However, C. albicans treated with xy12 could still grow slowly in nonfermenting carbon sources, which suggests that other antifungal mechanisms are involved in the antifungal action of xy12. In addition, our results showed that a higher concentration of xy12 was required to inhibit the proliferation of C. albicans cultured in nutrient-rich YPD medium, which may be related to the inhibitory effect of xy12 on oxidative phosphorylation. As we know, faster proliferation requires more energy and more active oxidative phosphorylation. When C. albicans proliferated rapidly, a higher concentration of xy12 was needed to inhibit active oxidative phosphorylation.

At the same time, the inhibition of mitochondrial complex I leads to defects in hypha and biofilm formation in C. albicans. The XTT assay used for biofilm detection reflects the activity of mitochondrial dehydrogenase, which may be affected by the antifungal mechanisms of xy12. Therefore, we also used crystal violet staining to clarify the inhibitory effect of xy12 on biofilm formation and obtained similar results. However, the inhibitory concentration of xy12 on hypha and biofilm formation was higher than the MIC_90_. Therefore, it is difficult to demonstrate that the inhibitory effects on hypha and biofilm were related to disrupted mitochondrial function or inhibition of proliferation. Currently, we have demonstrated that xy12 mainly exerts antifungal effects by inhibiting mitochondrial complex I. However, the research on the direct binding of xy12 with mitochondrial complex I was not in-depth. In the future, we will mainly focus on NADH dehydrogenation and coenzyme Q production occurring in mitochondrial complex I and identify the critical subunits which interact with xy12.

In conclusion, an effective and low-toxic antifungal compound, xy12, was obtained in our study through the modification of SM21. Compound xy12 could not only inhibit the proliferation of yeast cells, but also inhibit hypha and biofilm formation in C. albicans. Through comprehensive transcriptomic and metabolomic analysis, we found that xy12 mainly inhibited the mitochondrial oxidative phosphorylation pathway. The sensitivity of mitochondrial complex-deficient mutants and the effect of xy12 on ATP production indicated that xy12 mainly inhibited the function of mitochondrial complex I. Although the antifungal target of xy12 has not been elucidated, the changes in oxidative phosphorylation provide an important basis for subsequent exploration. Our study provides a novel lead compound for the development of mitochondria-targeted antifungals.

## MATERIALS AND METHODS

### Strains and culture conditions.

Clinical C. albicans strains UCA3, UCA21, UCA32, UCA42, UCA103, and UCA385 were isolated from patients with fungal infections in the Dermatology Department of Changhai Hospital (36). C. albicans strain SC5314 and mitochondrial complexes-deficient mutants (*aep1*Δ/Δ, *coe1*Δ/Δ, *coe2*Δ/Δ, *qce1*Δ/Δ, *nue*1Δ/Δ, *nue2*Δ/Δ, *nuo3*Δ/Δ, *nuo4*Δ/Δ, *pet111*Δ/Δ) were provided by William A. Fonzi (9). Strains were routinely cultured in YPD medium. Phosphate-buffered saline (PBS [pH 7.2]) (Sangon Biotech, Shanghai), Alcian blue (Sangon Biotech, Shanghai), rotenone and carboxin (Maclin Inc.), and ConA-FITC and XTT (Sigma) were used as indicated.

### MIC determinations.

C. albicans strains were incubated overnight in YPD liquid medium with 200 rpm oscillation at 30°C. The MICs of xy12, SM21 and fluconazole (FLC) against C. albicans were determined by the microdilution method as described by the Clinical and Laboratory Standards Institute (M27-A3) with a few modifications (37). Briefly, the cell suspensions of C. albicans were adjusted to 1 × 10^3^ to 3 × 10^3^ cells/mL in RPMI 1640 medium. Concentrations of xy12, SM21, and FLC from 16 to 0.0313 μg/mL were prepared in RPMI 1640 medium by a 2-fold dilution method. Concentrations of FLC from 64 to 0.125 μg/mL were prepared. Each dilution was added into a 96-well plate with C. albicans suspensions and incubated at 30°C for 24 h. RPMI 1640 medium used as the control. OD_600_ (optical densities at 600 nm) were determined by a microplate reader and the MIC was defined as the minimum concentration required to inhibit more than 90% of C. albicans growth.

### Disk-diffusion assay.

C. albicans SC5314 was incubated overnight and adjusted to 1 × 10^5^ cells/mL with PBS. Five mL of fungal suspension was plated on the YPD agar and incubated for 15 min. The supernatant was then removed, and the filter disks were plated on the agar. Five μL of antifungal drugs was spotted on each filter disk. The plates were then incubated at 30°C for 24 h and the inhibition zones were photographed.

### Time-growth and time-kill curves.

The time-growth curves were measured as described previously (38). C. albicans SC5314 was incubated overnight and washed with PBS. The concentration of C. albicans SC5314 was adjusted with YPD liquid medium to OD_600_ = 0.1. Different concentrations of xy12 were added based on the MIC_90_ values and the mixture was incubated at 30°C with 200 rpm oscillation. OD_600_ was measured at 0, 3, 6, 9, 12, and 24 h using a UV spectrophotometer and a curve was drawn to record the growth of C. albicans SC5314 at each time point.

The time-kill curves were examined as described previously (39). C. albicans SC5314 was cultured overnight and adjusted to 1 × 10^6^ cell/mL with YPD or RPMI 1640 medium. Strains were incubated with 0.5, 1, 2, or 4 μg/mL of xy12 for 6 or 12 h. After incubation at the indicated times, strains were collected and plated on Sabouraud dextrose agar and cultured for 48 h at 30°C. The live colonies were then counted.

### Biofilms formation assayed by XTT and crystal violet.

Biofilms formation was performed as described previously (40). C. albicans SC5314 was incubated overnight at 30°C with 200 rpm oscillation and washed with PBS. The concentration of C. albicans SC5314 was adjusted with RPMI 1640 medium to OD_600_ = 0.5. Next, 100 μL of fungal suspension was added to each well of a 96-well plate except for the negative control and cultured at 37°C for 90 min. Sterile PBS was then added to remove the non-adherent C. albicans. After this, different concentrations of xy12 diluted by fresh RPMI 1640 medium were added to the wells. The 96-well plate was then cultured at 37°C for 24 h and the biofilms were washed softly with PBS. Afterwards, 200 μL of XTT/menadione was added to the wells and incubated at 37°C for another 2 h. Finally, the absorbance of the supernatant was measured at 490 nm using a microplate reader. The ratio of biofilm formation was calculated as the OD_490_ of xy12-treated cells divided by the OD_490_ of untreated cells. For crystal violet staining, the biofilm-coated wells were established and treated with xy12 as described previously. Next, 100 μL of 0.4% aqueous crystal violet was added to the washed wells and stained for 45 min. Afterwards, each well was washed three times with 200 μL of sterile water and de-stained with 200 μL of 95% ethanol for 45 min. The absorbance of de-stained crystal violet at 600 nm was determined by a microplate reader. The condition of biofilm formation in the wells was observed under a microscope.

### Confocal microscopy.

C. albicans SC5314 or Tom70p-GFP strains were incubated overnight, and the concentrations were adjusted with YPD liquid medium to 1 × 10^6^ cells/mL. Different concentrations of xy12 and FLC, respectively, were added to the medium, and they were incubated at an oscillation rate of 200 rpm at 30°C for 6 h. Next, the C. albicans SC5314 strains were collected by centrifugation and stained with 100 nM MitoTracker Red CMXRos and imaged with a Leica confocal laser scanning microscope. The Tom70p-GFP strain was observed without MitoTracker staining.

### Mitochondrial membrane potential assay.

The JC-1 aggregates were used to determine the mitochondrial membrane potential as previously described (41). C. albicans SC5314 was incubated overnight and washed with PBS. The fungal cells were resuspended with fresh YPD medium supplemented with different concentrations of xy12 and incubated for 6 h at 30°C. Equal volumes of JC-1 staining solution and fungal fluid were mixed and stained for 20 min at 37°C in dark (Mitochondrial Membrane Potential Assay kit with JC-1, Beyotime). The fluorescence intensity (590 nm/530 nm) was determined by microplate reader.

### Transcriptome analysis.

RNA was extracted from the C. albicans SC5314 treated with dimethyl sulfoxide (DMSO) or 2 μg/mL of xy12 for 3 h using the column fungal RNAout kit (Tiandz). The quantity and quality of the extracted RNA were assessed using a Nanodrop 2000 (Thermo Fisher Scientific). The cDNA libraries were established and sequenced using the Illumina HiSeq platform by Applied Protein Technology (Shanghai, China). To obtain clean reads, low-quality reads (reads with Qphred ≤25 bases accounting for more than 60% of the entire reads) and ambiguous nucleotides (*n* ≥ 5%) were removed. PCA was performed with SIMCA version 14.1 using the quantitative data of the two omics. Differential analysis of expression was performed by DESeq2 and the differentially expressed genes were defined as *P* < 0.05 and |log_2_-foldchange| ≥ 1. GO and KEGG functional enrichment were analyzed by Fisher’s exact test to enrich the differentially expressed genes and biological functions.

RT-qPCR was performed with an ABI 7500 Real-Time PCR system. The primers used in this study are listed in Table S1. TaKaRa TB Green Extaq was used to amplify cDNA. Finally, the expression of each gene was normalized to the expression of *ACT1*. The relative expression of each gene was calculated by the threshold cycle (2^−ΔΔ^*^CT^*) method. The first Δ*C_T_* was calculated by the *C_T_* value of the target gene minus the *C_T_* value of the reference gene *ACT1* in each sample. The ΔΔ*C_T_* was calculated by the Δ*C_T_* value of the xy12-treated samples minus the Δ*C_T_* value of the DMSO-treated (mock) samples.

### Untargeted metabolomics analysis.

C. albicans SC5314 were collected by centrifugation and cryopreservation in liquid nitrogen after treatment with 2 μg/mL of xy12 for 8 h. Next, 50 mg wet weight of each sample was transferred to a 1.5-mL tube with 500 μL of extraction solution (methanol:acetonitrile:water = 2:2:1). Samples were vortexed and extracted by ultrasound for 30 min. Afterwards, each sample was incubated on ice for 10 min and centrifuged at 14,000 × *g* for 20 min at 4°C. The supernatants were dried in vacuum and dissolved with 100 μL of aqueous acetonitrile solution (acetonitrile:water = 1:1).

The supernatant of each sample was analyzed by UHPLC (Agilent 1290 Infinity LC) coupled to a quadrupole time-of-flight (AB Triple TOF 6600) from Shanghai Applied Protein Technology Co., Ltd. An ACQUITY UPLC BEH Amide column (1.7 μm, 2.1 mm × 100 mm; Waters Corporation, Milford, MA) was used with a column temperature of 25°C; the volume of injected samples was 2 μL and the flow rate was 0.5 mL/min. The components of mobile phase A were water, 25 mM ammonium acetate, and ammonia. The component of mobile phase B was acetonitrile. The chromatographic gradient elution protocol was as follows: 0 to 0.5 min, 95% B; 0.5 to 7 min, 95% to 65% B; 7 to 8 min, 65% to 40% B; 8 to 9 min, 40% B; 9 to 9.1 min, 40% to 95% B; 9.1 to 12 min, 95% B. The mass spectrometry electrospray ionization source settings were as follows: ion source gas 1 (Gas1), 60, ion source gas 2 (Gas2), 60; curtain gas (CUR), 30; source temperature, 600°C, IonSpray Voltage Floating, ±5,500 V; time of flight mass spectrometry (TOF MS) scan *m/z* range, 60 to 1,000 Da; product ion scan *m/z* range, 25 to 1,000 Da; TOF MS scan accumulation time, 0.20 s/spectra; product ion scan accumulation time, 0.05 s/spectra; polarity: positive, negative. Secondary mass spectrometry was acquired using information dependent acquisition (IDA) and high sensitivity mode, a declustering potential (DP) of ±60 V (both positive and negative modes), and a collision energy of 35 ± 15 eV. IDA settings were as follows: exclude isotopes within 4 Da, 10 candidate ions to monitor per cycle. The raw MS data (wiff.scan files) were converted to MzXML files. Available XCMS software was used to analyze total peak intensity and retention time. The processed data were analyzed by the R package “ropls,” where it was subjected to multivariate data analysis, including orthogonal partial least-squares discriminant analysis. The variable importance in the projection value of each variable in the OPLS-DA model was calculated to indicate its contribution to the classification. Metabolites with an OPLS-DA VIP value of >1 were further subjected to Student’s *t* test at the univariate level to measure the significance of each metabolite; *P* < 0.05 was considered statistically significant. The annotation of metabolites was performed by the KEGG databases (https://www.genome.jp/kegg/pathway.html).

### Combined transcriptome and metabolome analysis.

Combined analysis of RNA-seq and liquid chromatography-tandem mass spectrometry profiles were calculated with the Pearson algorithm in R version 3.5.1. All differentially expressed genes and metabolites were queried and mapped to pathways based on the online KEGG database (http://www.kegg.jp/). Enrichment analysis was also performed. R version 3.5.1 was used to combine the KEGG annotation and enrichment results.

### Spot assay.

C. albicans strains were incubated overnight at 30°C in liquid YPD medium. Each strain was diluted to 5-fold serial densities ranging from 1 × 10^7^ to 1.6 × 10^4^ cells/mL with PBS. Five μL of each fungal suspension was spotted on YPD with or without xy12 (0.25 or 0.5 μg/mL). The plates were cultured at 30°C for 48 h and photographed.

### Measurement of mitochondrial complex I activity.

The mitochondria of C. albicans were isolated as previously described with some modifications (42). Briefly, C. albicans SC5314 cells were washed twice with PBS. Cell pellets were incubated with 1 mL of buffer A (50 mM K_2_HPO_4_, 50 mM KH_2_PO_4_, 5 mM EDTA, 50 mM dithiothreitol, 40 mM β-mercaptoethanol, and 2 M sorbitol) containing 40 U zymolase to prepare protoplasts. After a 90-min incubation, cells were centrifuged (1,000 × *g* for 5 min) and washed with twice buffer B (0.6 M mannitol, 0.2% bovine serum albumin, and 10 mM imidazole). Cells were then suspended in 1 mL buffer B and homogenized with a hand-held glass homogenizer for 3 min on ice. The supernatant was collected by centrifugation (5 min at 1,000 × *g*). The crude mitochondria were obtained by centrifugation (20 min at 12,000 × *g*) and resuspended in the extraction buffer (Solarbio, Beijing, China). The activities of mitochondrial complexes I were determined by a Micro Mitochondrial Respiratory Chain Complex Activity assay kit (Solarbio, Beijing, China). Different concentrations of xy12 were added to the mitochondrial suspensions and they were incubated for 3 h at 30°C.

### Determination of ATP concentrations.

A BacTiter-Glo Microbial Cell Viability assay kit (Promega Corporation) was used to determine the ATP concentrations in C. albicans (43). C. albicans SC5314 was incubated overnight and washed three times with PBS. The density of fungal cells was diluted to 5 × 10^6^ cells/mL in YPD liquid medium with or without xy12 (2 or 4 μg/mL). C. albicans was then incubated for 6 h at 30°C. Next, 50 μL of fungal suspensions was vortexed with 50 μL of BacTiter-Glo Reagent and added to a 96-well plate for a 10-min incubation. The fluorescence intensity was determined by a microplate reader. ATP standard curves were drawn to calculate the ATP content as per the manufacturer’s instructions.

### Alcian blue binding.

Alcian blue binding was used to detect phosphomannan as described previously (44). The standard curve of Alcian blue was established using concentrations of 1.56 to 50 μg/mL. C. albicans SC5314 was treated with 2 or 4 μg/mL of xy12 for 16 h. Fungal cells were washed three times with PBS and adjusted to 1.5 × 10^7^ cells/mL. Next, 1 mL of fungal suspension was centrifuged at 8,000 × *g*. Cells were resuspended with 1 mL of Alcian blue (30 μg/mL) and incubated for 10 min at 30°C. After centrifugation, the supernatant was used to measure OD_600_. The amount of adsorbed Alcian blue was calculated by the standard curve.

### Data availability.

The RNA-seq data used in our study have been deposited to the China National Center for Bioinformation under accession no. CRA006711.
