# Supplementary material for: Combined Transcriptome and Metabolome Analysis Reveals That the Potent Antifungal Pyrylium Salt Inhibits Mitochondrial Complex I in Candida albicans
Source: Microbiol Spectr. 2023 Feb 15;11(2):e03209-22. doi: 10.1128/spectrum.03209-22 (PMC10100848; doi:10.1128/spectrum.03209-22)
Supplement: Supplemental file 1 — Supplemental material. Download spectrum.03209-22-s0001.pdf, PDF file, 0.5 MB [file spectrum.03209-22-s0001.pdf]

Table S1. Primers used in this study.

name	sequence	name	sequence
ACT1-F	TTGATTTGGCTGGTAGAGAC	NUO1-F	ACAGCTACTAATCAACCAGTGAG
ACT1-R	ATGGCAGAAGATTGAGAAGA	NUO1-R	CAAGTGGGAATGATGCCGTAA
NAD5-F	TACTTGAATTAGTCTAGGTG	NUO2-F	AAGAAGCACAGAATTACAAGGACC
NAD5-R	CTACAAACTCTCAACCAATG	NUO2-R	CACAGATGCAGGCACAGACTT
ATP6-F	TTCACCTTTAGATCAATTCG	NUO3-F	AGTTCTGTGTTTGGTGCATCT
ATP6-R	AAACTAAATGATACAATAGC	NUO3-R	GCAGTTGTGTGTTTTGGATGG
NUE1-F	TCGAATAACACCACCTCCCA	NUO4-F	TGCTTTCTGGAGTAAGACAACA
NUE1-R	CCAAGATTCATCACGACAGG	NUO4-R	CCCCAGTGGTGATTTCGTCA
NDH51-F	ATTAAGAGGTCGTGGAGGTGC	MNE1-F	GCCAAATAACATCAAGTCATCGT
NDH51-R	GCAGCAGTGGCATTCATAGC	MNE1-R	GGTGTTGTTGTTGTGAGTCTGA
orf19.4324-F	GGCACAACAAGCTGATGAGA		
orf19.4324-R	TGTTTCCAAGGATCTTCAACCC		

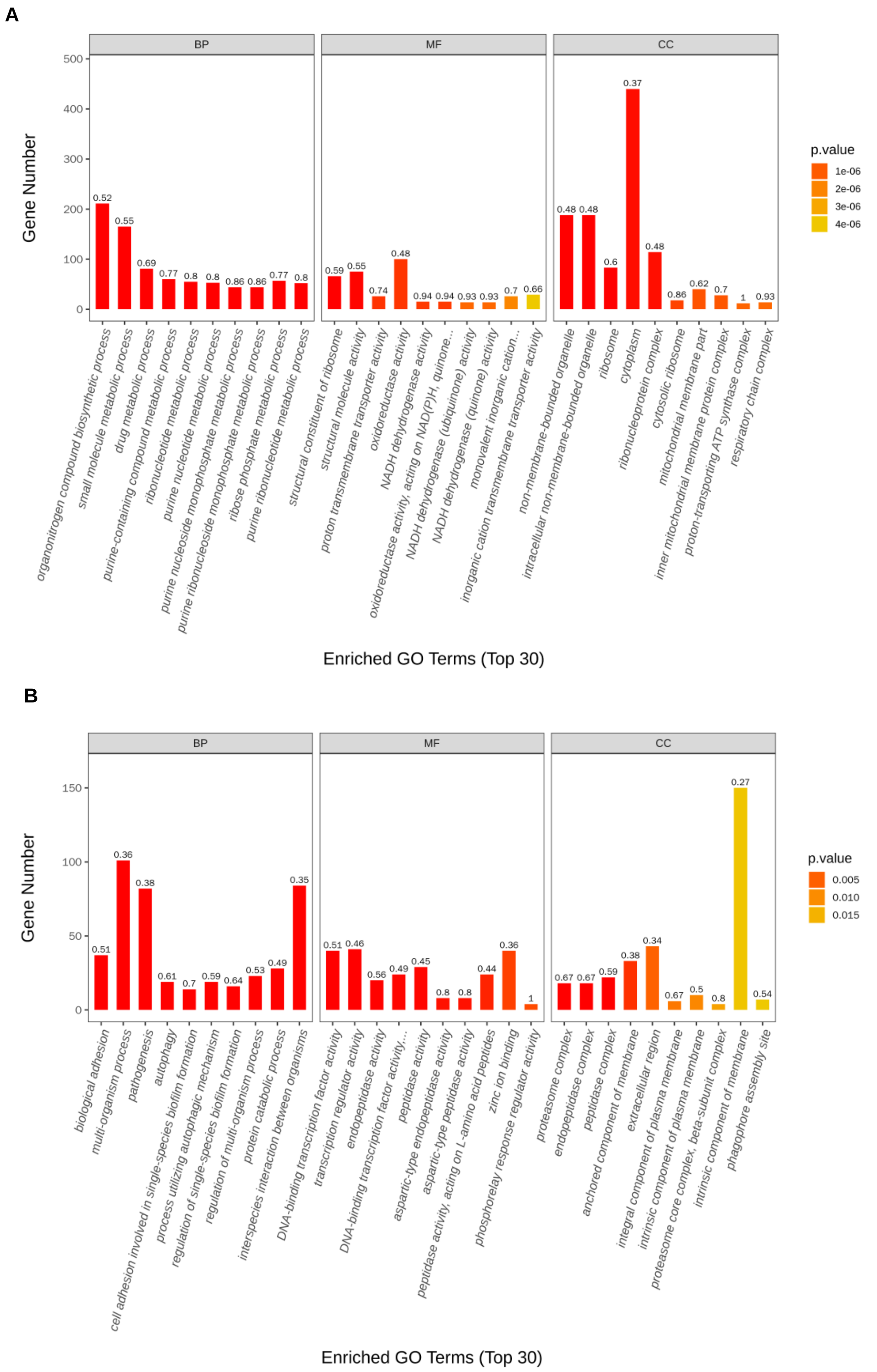

Fig. S1 Differentially expressed genes (DEGs) in xy12-treated *C. albicans*. GO terms analysis of down-regulated (A) and up-regulated (B) genes.

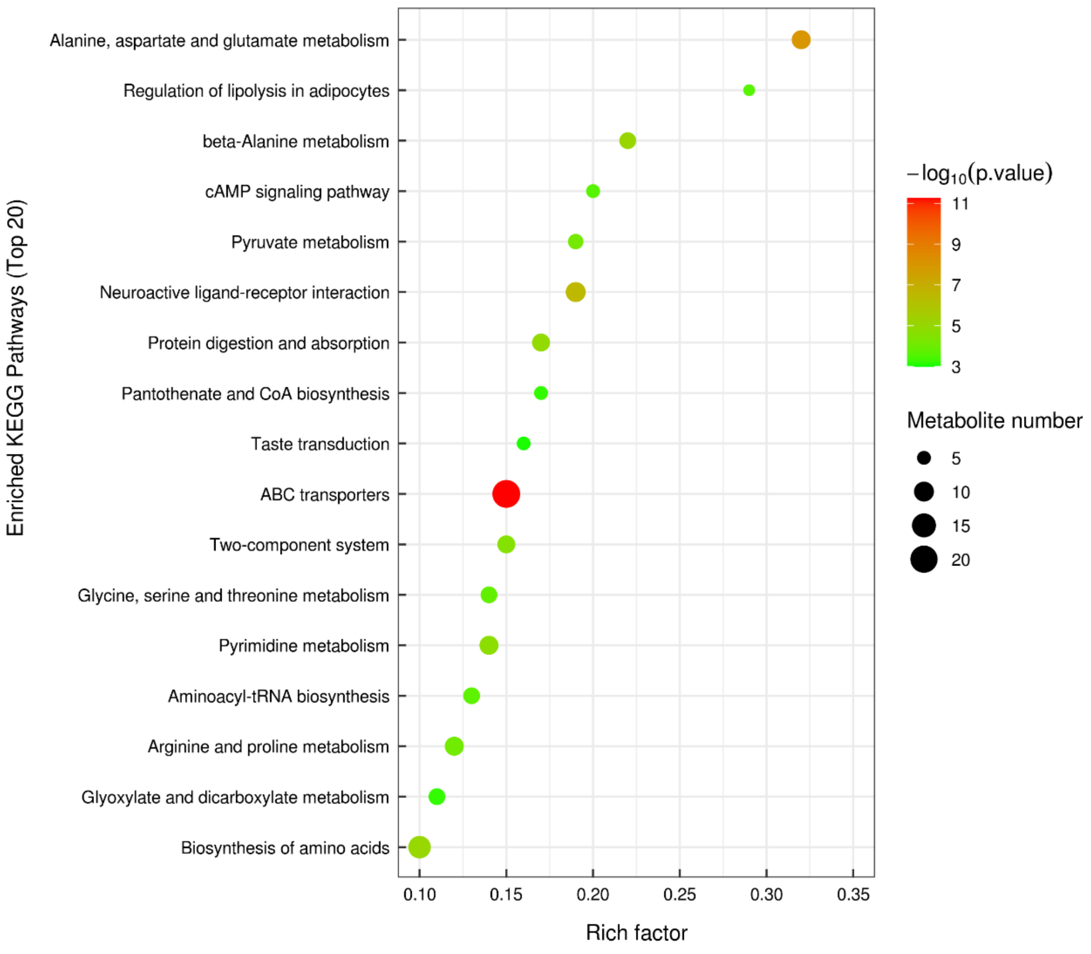

Fig. S2 The KEGG enrichment analysis of significantly different metabolites.
